# Once-Weekly Semaglutide Is Associated With Improvement in Vascular Endothelial Function in Patients With Type 2 Diabetes Mellitus: A Retrospective Observational Study

**DOI:** 10.7759/cureus.99998

**Published:** 2025-12-24

**Authors:** Seigo Sugiyama, Akira Yoshida, Noboru Kurinami, Kunio Hieshima, Katsunori Jinnouchi, Tomoko Suzuki, Fumio Miyamoto, Keizo Kajiwara, Hideaki Jinnouchi

**Affiliations:** 1 Diabetes Care Center, Jinnouchi Hospital, Kumamoto, JPN

**Keywords:** cardiovascular diseases, glucagon-like peptide-1 receptor agonist, japanese population, semaglutide, type 2 diabetes mellitus, vascular endothelial function

## Abstract

Background: Patients with type 2 diabetes mellitus (T2DM) have a high-risk condition of developing cardiovascular disease, and therefore, therapeutic strategies should consider cardiovascular risk reduction. Previously, we demonstrated that circulating glucagon-like peptide-1 (GLP-1) levels are significantly lower in Japanese patients with coronary artery disease (CAD) compared with non-CAD subjects. Once-weekly semaglutide, a GLP-1 receptor agonist, has demonstrated clinical cardiometabolic benefits; however, its effect on vascular endothelial function in Japanese patients with T2DM remains unclear.

Methods: This retrospective observational study included patients with T2DM who were hospitalized at Jinnouchi Hospital and initiated once-weekly subcutaneous semaglutide therapy since 2021. Vascular endothelial function was assessed using reactive hyperemia peripheral arterial tonometry, expressed as the reactive hyperemia index (RHI), before and after semaglutide treatment. Clinical parameters including body weight (BW), glycated hemoglobin A1c (HbA1c), low-density lipoprotein cholesterol (LDL-cho), and high-sensitivity C-reactive protein (hsCRP) were also evaluated.

Results: A total of 24 patients (mean age: 55 years; n=20 (83.3%) male) were included. RHI significantly increased following semaglutide therapy (baseline: 1.62±0.19 versus follow-up: 2.04±0.60; p<0.01). BW, HbA1c, and LDL-cho also showed significant reductions (all p<0.01). HsCRP tended to decrease, though not significantly. None of the changes in BW, HbA1c, LDL-cho, or hsCRP correlated with the changes in RHI.

Conclusion: Once-weekly semaglutide treatment significantly provided potential improvement in vascular endothelial function in Japanese patients with T2DM, supporting the cardiovascular protective role of semaglutide therapy in the clinical management of T2DM.

## Introduction

Type 2 diabetes mellitus (T2DM) is associated with a markedly increased risk of cardiovascular disease (CVD) [1]. Therefore, clinical management strategies should consider the prevention of cardiovascular events in addition to glycemic control [2]. Previously, we revealed that circulating glucagon-like peptide-1 (GLP-1) levels are significantly lower in Japanese patients with coronary artery disease (CAD) compared with non-CAD subjects [3]. GLP-1 receptor agonists (GLP-1 RAs) have demonstrated multiple cardiometabolic benefits including body weight (BW) reduction, improved glycemic control and insulin resistance, and reduction in cardiovascular event risk [4-7]. Semaglutide is a once-weekly subcutaneously administered GLP-1 RA with demonstrated efficacy in global cardiovascular outcome trials [8]. It also showed the effects of semaglutide on CVD risk reduction especially among Asian populations [8]; however, the cardiovascular benefits have not been well clarified in the Japanese population [9,10].

Vascular endothelial dysfunction is a physiological marker of atherosclerosis and a predictor of future cardiovascular events [11]. The reactive hyperemia peripheral arterial tonometry (RH-PAT) test provides a noninvasive and reproducible assessment of endothelial function by quantifying the reactive hyperemia index (RHI) [12]. Although GLP-1 RAs have been suggested to improve endothelial function via anti-inflammatory and antioxidative mechanisms [13], evidence specific to semaglutide in Japanese T2DM patients is limited [8,10].

This study aimed to evaluate the impact of once-weekly semaglutide on vascular endothelial function assessed by RH-PAT in patients with T2DM treated at a single Japanese center. The primary outcome was semaglutide therapy-induced changes in RHI values, and the secondary outcome was the correlation between changes in RHI and changes in metabolic and inflammatory markers.

This article was partly presented as a meeting abstract at the 63rd Kyushu Regional Meeting of the Japan Diabetes Society on November 1, 2025. The abstract was posted in Japanese.

## Materials and methods

Study population and study protocol

This single-center, retrospective observational study included adult patients diagnosed with T2DM who were hospitalized at Jinnouchi Hospital in Kumamoto, Japan, between January 2021 and December 2024 and initiated once-weekly subcutaneous semaglutide therapy (0.25 mg/week). Patients were included if RH-PAT measurements were performed both before and after the initiation of semaglutide. Follow-up RH-PAT assessments were performed at least six months after baseline, with a median follow-up duration of nine months (interquartile range (IQR) 6-12 months). Patients were followed monthly, during which the attending physician evaluated glycated hemoglobin A1c (HbA1c), glucose levels, BW, and patient preferences and determined semaglutide dose adjustments accordingly. Patients with cancer, severe infection, recent cardiovascular events, heart failure (New York Heart Association Class III or higher), advanced chronic kidney disease, advanced hepatic diseases, or concomitant use of other GLP-1 RAs were excluded. We conducted the present retrospective study in accordance with the Declaration of Helsinki. The Human Ethics Review Committee of Jinnouchi Hospital approved the present study protocol (approval number: 2025-6-(1)), and we obtained a signed informed consent form from each patient. We registered the present study protocol in the University Hospital Medical Information Network (UMIN) protocol registration system (UMIN: 000068149).

Endothelial function assessment

Previously, the details of the RH-PAT test have been reported [14,15]. We clinically examined vascular endothelial function in the fingertip by RH-PAT using the Endo-PAT2000® (Itamar Medical, Caesarea, Israel). The results of the RH-PAT test were provided as RHI values. The RH-PAT test evaluates endothelial-dependent vasodilation by measuring pulse wave amplitude changes in the fingertip before and after transient arterial occlusion. The RHI is automatically calculated as the ratio of post-occlusion to baseline pulse wave amplitude, normalized to the control arm. Higher RHI values indicate better endothelial function. The reproducibility of RH-PAT measurements has been validated in previous studies, demonstrating good inter-session reliability with intraclass correlation coefficients ranging from 0.60 to 0.78 and low day-to-day variability [14,16]. These data support the use of RHI as a robust and reproducible index of endothelial function. We performed the tests when patients were in a stable and fasting condition in the morning without taking their daily medications.

Measurement of blood parameters and assessment of clinical characteristics

We collected fasting blood from the antecubital vein in the morning, and the blood parameters were analyzed at the outpatient's laboratory in Jinnouchi Hospital to assess the values of HbA1c, fasting plasma glucose (FPG), postprandial plasma glucose (PPG), cholesterols, triglycerides, and high-sensitivity C-reactive protein (hsCRP). BW, height, body mass index (BMI), lipid profiles, blood pressure, heart rate, and clinical past history were recorded.

The primary and secondary outcomes

The primary outcome was semaglutide therapy-induced changes in RHI values. We investigated the semaglutide-induced changes in RHI values compared with the baseline ones. The secondary outcome was the correlation between changes in RHI values and changes in HbA1c, BW, low-density lipoprotein cholesterol (LDL-cho), and hsCRP.

Statistical analysis

We performed a normal distribution test for continuous data using the Shapiro-Wilk test, and the normally distributed data was expressed as the mean (standard deviation (SD)), while those of continuous data with a skewed distribution are shown as median values (IQR). We applied either the paired Student's t-test or the Wilcoxon test to analyze the effect of the semaglutide therapy. We used the Mann-Whitney U test to compare the RHI values between CAD and non-CAD patients. We determine the relationships between changes in HbA1c, BW, LDL-cho, and hsCRP and changes in RHI values, using Pearson's correlation coefficient. Statistical significance was defined as p<0.05. We performed all statistical analyses using IBM SPSS Statistics for Mac, V. 23.0 (IBM Corp., Armonk, NY, USA).

## Results

Baseline characteristics, medications, and follow-up duration

We finally included 24 Japanese stable patients with T2DM. Table 1 indicates the clinical baseline characteristics of the total patients.

**Table 1:** Baseline clinical characteristics Values of age, body weight, and body mass index are presented as mean (±SD). Values of sex, hypertension, dyslipidemia, current smoking, retinopathy, and cardiovascular disease are presented as the number of patients (%). Values of duration of diabetes, hemoglobin A1c, and fasting plasma glucose are presented as median (IQR). IQR: interquartile range; SD: standard deviation

	Total subjects (n=24)
Age (year)	55.1±13.1
Sex: male (%)	20 (83.3%)
Body weight (kg)	78.1±13.7
Body mass index (kg/m^2^)	27.9±2.9
Hypertension (%)	15 (62.5%)
Dyslipidemia (%)	24 (100%)
Current smoking (%)	11 (45.3%)
Retinopathy (%)	6 (25%)
Cardiovascular diseases (%)	5 (20.3%)
Duration of diabetes (years)	7 (3-11)
Hemoglobin A1c (%)	7.9 (7-9.4)
Fasting plasma glucose (mg/dL)	133 (123-160)

The patients were 55 years old in mean and male (n=20; 83.3%), with a BMI of 27.9 kg/m^2^ (mean). The median values of HbA1c and FPG were 7.9% and 133 mg/dL. At baseline condition, eight patients (33.3%) were treated with insulin, and 12 patients (50%) were treated with metformin. Twenty-one patients (87.5%) were already receiving sodium-glucose cotransporter-2 (SGLT2) inhibitors at baseline (Table 2). The median duration of diabetes was 7.9 years. The patients were followed for nine (six to 12) months in median and (IQR). The average dose of semaglutide was 0.35 mg.

**Table 2:** Baseline medical therapy All data are presented as the number of patients (%). CCB: calcium channel blockers; ACEi: angiotensin-converting enzyme inhibitors; ARB: angiotensin II receptor blockers

	Total subjects (n=24)
Glucose-lowering medication	-
Sulfonylurea (%)	0 (0%)
Glinide (%)	3 (12.5%)
Metformin (%)	12 (50%)
Alpha-glucosidase inhibitor (%)	0 (0%)
Thiazolidinedione (%)	0 (0%)
Dipeptidyl peptidase-4 inhibitor (%)	0 (0%)
Imeglimin (%)	0 (0%)
Sodium-glucose cotransporter-2 inhibitor (%)	21 (87.5%)
Insulin (%)	8 (33.3%)
CCB or ACEi or ARB (%)	9 (37.5%)
Statin (%)	24 (100%)

Vascular endothelial function assessed by RH-PAT before and after semaglutide therapy

**Figure 1:**
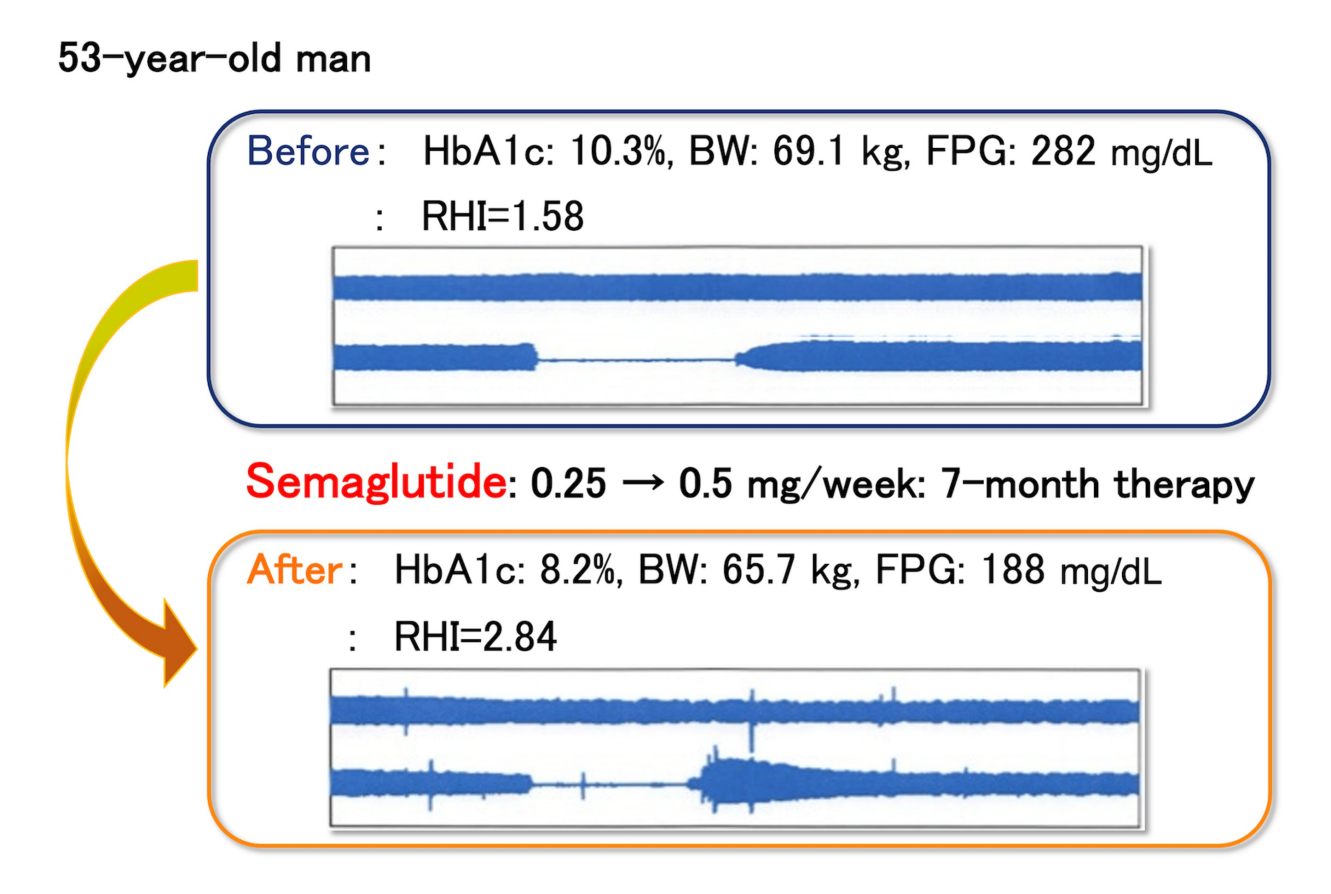
Representative changes in endothelial function as evaluated by an RH-PAT test in semaglutide therapy A 53-year-old man was treated with once-weekly semaglutide for seven months. Semaglutide was initially given at a dosage of 0.25 mg, and, after one month, the dose was increased to 0.5 mg and continued. The representative record of the reactive RH-PAT examination demonstrated the increase in the early response to reactive hyperemia. HbA1c: glycated hemoglobin A1c; BW: body weight; FPG: fasting plasma glucose; RHI: reactive hyperemia index; RH-PAT: hyperemia peripheral arterial tonometry

**Figure 2:**
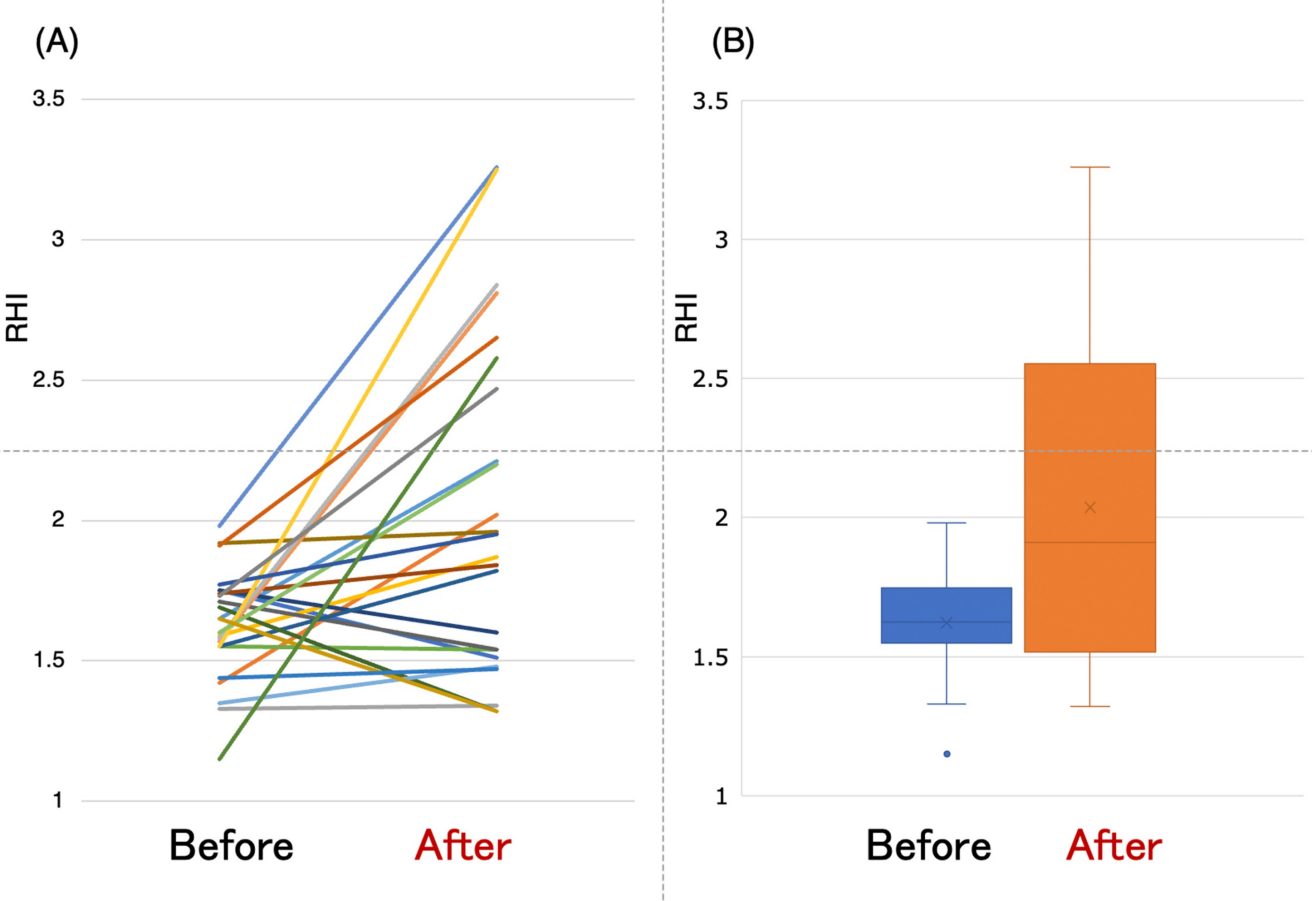
The line graph (A) and the box and whisker graph (B) demonstrated the change in endothelial function assessed by RHI in each patient before and after the semaglutide therapy RHI: reactive hyperemia index

Figure 1 demonstrates the representative records of RH-PAT examinations before and after the semaglutide therapy. The vascular endothelial function assessed by RHI significantly increased after semaglutide therapy (baseline: 1.62±0.19 vs. follow-up: 2.04±0.60; p<0.01; Figure 2). Regarding the ΔRHI difference between CVD (n=5) and non-CVD (n=19) patients, the median ΔRHI did not differ significantly between groups (CVD: 0.280 vs. non-CVD: 0.225; Mann-Whitney U test=63.5; p=0.862).

Changes in BW, BMI, blood pressure, heart rate, HbA1c, FPG, lipids, and hsCRP

As shown in Table 3, BW, BMI, LDL-cho, triglyceride, and HbA1c significantly decreased after treatment (p<0.01). HsCRP tended to decrease but was not statistically significant.

**Table 3:** Changes in body weight, blood pressure, and metabolic parameters Values of body weight, BMI, s-BP, heart rate, and LDL-cho are presented as mean (±SD). Values of HbA1c, FPG, HDL-cho, TG, AST, ALT, and hsCRP are presented as median (IQR). The p-values ​​(p<0.05) considered as significant are indicated by asterisk (*). The paired Student's t-test is applied for body weight, BMI, s-BP, heart rate, and LDL-cho. The Wilcoxon test is applied for HbA1c, FPG, HDL-cho, TG, AST, ALT, and hsCRP. BMI: body mass index; s-BP: systolic blood pressure; HbA1c: glycated hemoglobin A1c; FPG: fasting plasma glucose; HDL-cho: high-density lipoprotein cholesterol; LDL-cho: low-density lipoprotein cholesterol; TG: triglyceride; AST: aspartate aminotransferase; ALT: alanine aminotransferase; hsCRP: high-sensitivity C-reactive protein; IQR: interquartile range; SD: standard deviation

n=24	Baseline	After semaglutide therapy	Test statistics numerical value	P-value
Body weight (kg)	78.1±13.7	73.2±13.6	t=3.882	<0.001*
BMI (kg/m^2^)	27.9±2.9	26.1±3.4	t=3.997	<0.001*
s-BP (mmHg)	124.0±13.5	126.8±15.9	t=-1.008	0.324
Heart rate (beats/min)	76.3±10.2	77.8±8.7	t=-1.274	0.215
HbA1c (%)	7.9 (7-9.4)	6.1 (5.8-7.1)	Z=-3.771	<0.001*
FPG (mg/dL)	133 (123-160)	110 (96-118)	Z=-3.143	0.002*
HDL-cho (mg/dL)	45 (40-47)	53 (48-59)	Z=-3.657	<0.001*
LDL-cho (mg/dL)	100.5±33.7	69.1±19.1	t=4.585	<0.001*
TG (mg/dL)	136 (102-197)	97 (64-114)	Z=-3.571	<0.001*
AST (U/L)	27 (20-33)	19 (17-23)	Z=-3.319	<0.001*
ALT (U/L)	35 (21-49)	23 (18-27)	Z=-2.357	0.018*
hsCRP (pg/mL) (n=21)	0.09 (0.06-0.17)	0.04 (0.02-0.08)	Z=-1.946	0.051

Correlation between changes in HbA1c, BW, LDL-cho, and hsCRP and changes in RHI

**Figure 3:**
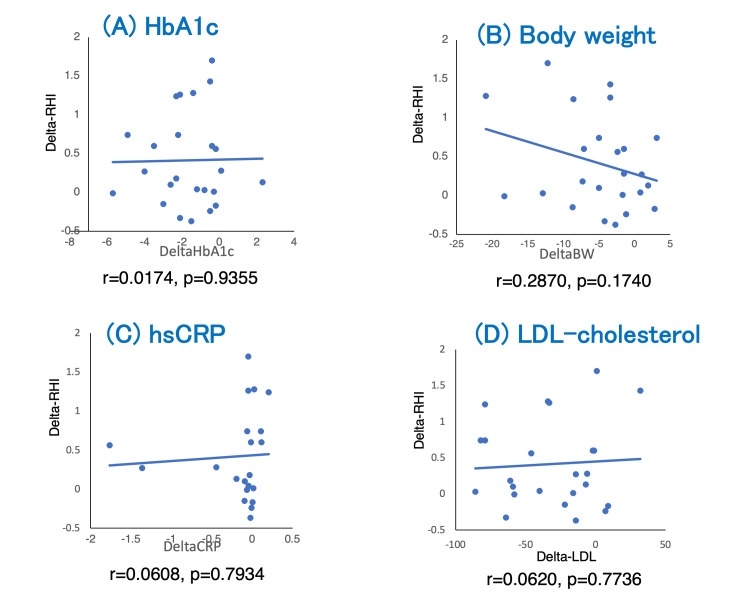
Dots plot graph demonstrated the correlation between changes in RHI and changes in HbA1c (A), body weight (B), hsCRP (C), and LDL-cholesterol (D) RHI: reactive hyperemia index; HbA1c: glycated hemoglobin A1c; hsCRP: high-sensitivity C-reactive protein; LDL-cholesterol: low-density lipoprotein cholesterol

As shown in Figure 3, we found a non-significant correlation between the change in RHI and the changes in BW, HbA1c, LDL-cho levels, and hsCRP levels.

## Discussion

In this retrospective analysis, once-weekly semaglutide is significantly associated with improvement in vascular endothelial function in Japanese patients with T2DM, as assessed by RH-PAT. Enhancement of endothelial function may contribute to the vasculoprotective properties of semaglutide.

Once-weekly semaglutide (Ozempic®) is recommended in the American Diabetes Association guidelines for patients with T2DM who have established CVD or are at high cardiovascular risk [17]. Moreover, semaglutide therapy is considered potentially effective for cardiovascular risk reduction among Asian individuals [8]; however, its clinical cardiovascular effects in Japanese populations remain insufficiently characterized, and further clinical research is warranted.

Our findings provide supportive evidence that semaglutide may provide potential enhancement in vascular endothelial health in Japanese patients, a mechanism potentially contributing to long-term cardiovascular benefit. Furthermore, as we previously and firstly demonstrated, active circulating GLP-1 levels are significantly decreased in Japanese patients with CAD compared to non-CAD ones [3]. This finding suggests that impaired endogenous GLP-1 signaling may contribute to the development of CVD and endothelial dysfunction in the Japanese population. Therefore, the significant increase in RHI as the index of endothelial function observed with semaglutide therapy in the present study may reflect, at least in part, the restoration of GLP-1-dependent vascular protective pathways. Given that baseline GLP-1 activity may be lower in Japanese CAD patients [3], GLP-1 RA treatment could potentially exert greater cardiovascular benefits in this population. These observations could reinforce the biological plausibility of cardiovascular protective effects of GLP-1 RA in Japanese patients with T2DM.

In the interpretation of RH-PAT measurements, it is important to note that no universally accepted clinical threshold defines what constitutes a "clinically significant" improvement in RHI. Previous interventional and observational studies have often considered an increase of approximately 0.3-0.4 in RHI as reflecting a meaningful enhancement of endothelial function, although this value has not been formally established as a clinical criterion [15,18]. In addition, earlier cross-sectional research proposed that RHI <1.67 indicates endothelial dysfunction, a cutoff that has been widely used in subsequent RH-PAT studies [19]. Furthermore, Matsuzawa et al. indicated that prospective data from a large cohort demonstrated the prognostic relevance of RH-PAT parameters [20], a 0.1 increase in natural logarithm of the reactive hyperemia index (Ln_RHI) was associated with a significantly reduced risk of cardiovascular events (adjusted relative risk 0.79; 95% CI: 0.71-0.87), and a 1-SD worsening in endothelial function doubled cardiovascular risk. These findings suggest that even modest changes in RHI may have clinical significance, although consensus thresholds for individual-level clinical decision-making remain to be established.

Previous studies have reported beneficial vascular effects of GLP-1 RAs through anti-inflammatory and antioxidative pathways, enhanced nitric oxide bioavailability, and reduced oxidative stress [13,21,22]. The absence of correlation between RHI increase and BW, HbA1c, or LDL-cho reduction suggests that the endothelial benefits may occur through mechanisms distinct from metabolic control. Possible mechanisms include GLP-1 receptor-mediated activation of endothelial nitric oxide synthase and attenuation of vascular oxidative stress [23-26]. Consistent with our clinical findings, preclinical studies report direct and indirect vascular protective effects of semaglutide. In murine atherosclerosis models, semaglutide reduced plaque burden [27], decreased inflammation in atherosclerosis [28], and attenuated post-injury vessel remodeling [29], while proteomic analyses in diet-induced obese mice indicate protective changes in vascular extracellular matrix components [30]. In vitro/ex vivo work further suggests semaglutide can protect endothelial progenitor cells via the modulation of macrophage-derived exosomal miR-155 [31]. These preclinical data provide mechanistic plausibility for the RHI increase we observed, although clinical causality requires further investigation.

In the present study, SGLT2 inhibitor use was nearly universal among the study population. SGLT2 inhibitors themselves have been known to show cardiovascular benefits; the uniform use of SGLT2 inhibitors in our study suggests that the observed improvement in RHI is unlikely to be explained by differences in SGLT2 inhibitor exposure.

Although RHI significantly increased after semaglutide therapy, the retrospective observational design and absence of a control group prevent the definitive attribution of the improvement solely to semaglutide. Therefore, the conclusions should be interpreted with caution.

Limitations

This study has several limitations. First, its retrospective design in a single center and a small sample size raises the possibility of selection bias in patient inclusion. Second, semaglutide dosing was not uniform, and dose variability may have affected treatment responses. Third, because the study was conducted without a comparator group, causal inference remains limited. Finally, as an exploratory and preliminary analysis, our findings should be confirmed in larger prospective controlled trials. There is a limitation regarding the generalizability of the present results, as our findings primarily reflect diabetes mellitus patients already treated with contemporary cardioprotective therapy including SGLT2 inhibitors and statins. We acknowledge that the very small sample size of the CVD group substantially limits the statistical power of the comparison between CVD and non-CVD groups. Therefore, the absence of statistical significance does not imply the absence of a true difference.

## Conclusions

Once-weekly semaglutide is associated with significant improvement in vascular endothelial function in Japanese patients with T2DM. The semaglutide-induced enhancement of endothelial function was not correlated with metabolic and inflammatory variables. These results may partly explain the beneficial effects of semaglutide on CVD-event prevention and support semaglutide as a promising vascular therapeutic option for cardiovascular risk mitigation in clinical diabetes management.
